# Effect of different root endophytic fungi on plant community structure in experimental microcosms

**DOI:** 10.1002/ece3.2416

**Published:** 2016-10-18

**Authors:** Carlos A. Aguilar‐Trigueros, Matthias C. Rillig

**Affiliations:** ^1^ Plant EcologyInstitut für BiologieFreie Universität BerlinBerlinGermany; ^2^ Berlin‐Brandenburg Institute of Advanced Biodiversity ResearchBerlinGermany

**Keywords:** endophytic *Fusarium*, plant community structure, root endophytic fungi

## Abstract

Understanding the effects of root‐associated microbes in explaining plant community patterns represents a challenge in community ecology. Although typically overlooked, several lines of evidence point out that nonmycorrhizal, root endophytic fungi in the Ascomycota may have the potential to drive changes in plant community ecology given their ubiquitous presence, wide host ranges, and plant species‐specific fitness effects. Thus, we experimentally manipulated the presence of root endophytic fungal species in microcosms and measured its effects on plant communities. Specifically, we tested whether (1) three different root endophyte species can modify plant community structure; (2) those changes can also modified the way plant respond to different soil types; and (3) the effects are modified when all the fungi are present. As a model system, we used plant and fungal species that naturally co‐occur in a temperate grassland. Further, the soil types used in our experiment reflected a strong gradient in soil texture that has been shown to drive changes in plant and fungal community structure in the field. Results showed that each plant species responded differently to infection, resulting in distinct patterns of plant community structure depending on the identity of the fungus present. Those effects depended on the soil type. For example, large positive effects due to presence of the fungi were able to compensate for less nutrients levels in one soil type. Further, host responses when all three fungi were present were different from the ones observed in single fungal inoculations, suggesting that endophyte–endophyte interactions may be important in structuring plant communities. Overall, these results indicate that plant responses to changes in the species identity of nonmycorrhizal fungal community species and their interactions can modify plant community structure.

## 1 Introduction

Understanding the drivers of community structure represents a major goal in plant community ecology. Theory predicts that community structure depends on species‐specific differential responses to strong abiotic and biotic factors (Chesson, 2000; Sarr, Hibbs, & Huston, 2005). At local scales, where climatic conditions are considered to be homogenous, species‐specific responses to changes in soil abiotic parameters are considered a major factor in determining the identity, relative abundance, and fitness of interacting plants (Wijesinghe, John, & Hutchings, 2005).

However, it is acknowledged that the composition of soilborne fungal symbionts strongly influence plant community structure as well. For example, experimental manipulations have shown strong plant responses to arbuscular mycorrhizal fungal (AMF) composition and diversity (van der Heijden, Boller, & Sanders, 1998; van der Heijden, Klironomos, et al., 1998). Moreover, evidence from soil feedback experiments in grasslands points out that other fungi than AMF could have similar effects on plant community dynamics (Bever et al., 2010; van der Heijden, Bardgett, & van Straalen, 2008).

Specifically, root endophytic fungi (REF) in the Ascomycota are a group of symbiotic partners that may be important players driving plant community structure in grassland ecosystems (Aguilar‐Trigueros, Powell, Anderson, Antonovics, & Rillig, 2014; Rodriguez, White, Arnold, & Redman, 2009). Because the term endophyte is ambiguous (it does not describe any phylogenetic or clearly defined functional group) (Aguilar‐Trigueros et al., 2014), here we use the term to refer to fungi that during root tissue colonization of grasses and forbs: (1) do not induce symptoms of disease; (2) do not form specialized fungal‐plant interfaces for the exchange of resources like the ones observed in AMF (the other major root fungal symbiont in grassland ecosystems). REF in grasslands occur in all major fungal phyla, but in comparison with AMF, there is less information on their influence on host physiology and their ecological role. A notable exception is REF in the Sebacinales (Basidiomycota), which have received substantial attention given their benefits conferred to their host (Weiss, Waller, Zuccaro, & Selosse, 2016); however, most evidence comes from studies on agricultural crops (Franken, 2012), remaining little understood their ecological role in the grasslands from which they have been isolated. However, there are several lines of evidence that indicate that REF in the Ascomycota may influence plant community structure.

First REF in the Ascomycota are abundant and ubiquitous, as revealed by community surveys using DNA sequencing methods (in some cases being five times higher in terms of species richness compared to AMF among grassland species; Wehner et al., 2014). Second, they can have broad host ranges (Hersh, Vilgalys, & Clark, 2011; Malcolm, Kuldau, Gugino, & Jimenez‐Gasco Mdel, 2013) while colonized plants species respond differently to even the same fungal genotype. For example, when multiple plant species were inoculated with the same strain of *Microdochium* sp. under the same conditions, some plants species showed increased biomass production while others remained unaffected despite being also colonized (Mandyam, Fox, & Jumpponen, 2012), whereas a strain of *Gibberella* sp pathogenic on *Pinus radiata* has been found as an endophyte among neighboring grasses without inducing disease (Swett & Gordon, 2012).

In fact, it is increasingly acknowledged that fungal species in the Ascomycota only described as pathogens actually live as asymptomatic endophytes on a larger set of hosts (Malcolm et al., 2013; Stergiopoulos & Gordon, 2014). However, their influence on these hosts as endophytes and subsequent effects on plan community structure are less studied.

A first step toward an empirical understanding of the ecological role of REF requires measuring their effects on plant communities in the absence of other interactions. With this information, subsequent studies can add more interactions (complexity) that reflect more realistic scenarios. This type of reductionist approaches has been successfully used in the past to determine the role of plant–plant competition (Freckleton & Watkinson, 2001) or plant–AMF interactions (van der Heijden, Boller, et al., 1998) in structuring plant communities.

Following this logic, in this study, we aimed at measuring the effect of different REF species on a natural grassland excluding other type of interactions. As manipulating REF species in the field is unpractical, we opted for a greenhouse microcosm study where we mimic the conditions found in a natural grassland in north eastern Germany. To construct the microcosm, we used REF in the Ascomycota that were isolated from an abundant plant in that grassland and whose genera are mostly known because of their pathogenic species.

In our microcosms, we specifically tested whether the different REF can modify the outcome of plant–plant interactions. We hypothesized that fungi could alter the growth of the host from which the fungi were isolated as well as of neighboring interacting species, thus modifying plant growth patterns that resulted from plant–plant competition in our microcosms. We added complexity to our system by further testing whether such effects depended on (1) soil type or (2) when all REF were present.

## 2 Materials and Methods

### 2.1 The study system

Our experimental microcosms were designed to recreate conditions found in a natural grassland located near the town of Mallnow, Lebus (Brandenburg, Germany, 52°127.77′N, 14°129.349′E) (referred as the “Mallnow Grassland” throughout this study). Specifically, this study is based on the vegetation occurring in a 15 × 15 m plot within this grassland that was used in previous studies in our laboratory (Horn, Caruso, Verbruggen, Rillig, & Hempel, 2014; Horn et al., 2015). Within the plot, there is a strong gradient in soil texture from loamy to sandy soil along a hillside. This variation in soil texture also corresponded with a steep change in soil parameters such as C/N ratio, available P and pH (Horn et al., 2014). Further, *Festuca brevipila* R. Tracey. was found to represent up to 70% of vegetation cover, which included 47 other herbaceous species, such as *Arrhenatherum elatius* (L.) P. Beauv. ex J. Presl & C. Presl., *Armeria elongata* Hoff., and *Rumex acetosella* L. (Horn et al., 2015), which have been frequently reported for dry grasslands in north eastern Germany (Hensen, 1997).

### 2.2 Fungal isolation and characterization

On October 2010, we dug out 27 plant individuals of the grass *F. brevipila* within a 15 × 15 m plot in the Mallnow Grassland. This plot was previously established to study AMF community structure in the grassland (Horn et al., 2014). The root systems of the 27 individuals of *F. brevipila* were sectioned into 1 cm (centimeters)‐long pieces, surface sterilized by submerging them into 0.525% sodium hypochlorite for 3 min and subsequently in 70% ethanol for 1 min, and finally washed six times with sterile water. Then, the root fragments were plated on malt extract agar (MEA 2%) with Rose Bengal. This protocol is frequently used to for the isolation of fungi out of root tissue (Crous, Verkley, Groenewald, & Samson, 2009).

We obtained a total of 258 isolates, which we initially grouped into morphotypes according to colony characteristics in pure culture. However, given this technique can be subjective, we opted to group the fungi based on RFLP patterns as a more accurate way to discriminate among different genera. To this end, we took a piece of mycelium from clean cultures of each isolate for DNA extraction using the Power Soil DNA isolation kit (MoBio Laboratories Inc., Carlsbad, CA, USA) following the procedures in the manufacturer′s manual. Then, we amplified the ITS region using the primers ITS1F (CTTGGTCATTTAGAGGAAGTAA) (Gardes & Bruns, 1993) and ITS4 (TCCTCCGCTTATTGATATGG) (White, Bruns, Lee, & Taylor, 1990). PCR products were digested for 2 hr at 37°C using the restriction enzymes *BsuRI*,* Hin61*,* HinfI*, and *MboI*. These enzymes have been used previously for screening of soilborne fungi (Viaud, Pasquier, & Brygoo, 2000). The digestion products were electrophoresed in 2% agarose gels, and RFLP profiles were then used to group morphotypes, resulting in 31 RFLP groups. We then selected at least one isolate per RFLP group for further sequencing. The PCR products of each RFLP type were purified to remove nonincorporated ITS primers using a PCR cleanup kit (Macherey‐Nagel, Düren, Germany) and sent to LGC Genomics (Berlin, Germany) for Sanger sequencing. Sequences were then used in a BLAST search to assign putative taxonomic affiliation (see Table S2 for the number of isolates and RFLP groups per plant individual).

For this study, we used ascomycetous fungi which the closest BLAST matches were *Fusarium redolens* (accession number GU934525.1, 100% identity match), *Gibberella* sp. (accession number JF773634.1, 100% identity match), and *Microdochium* sp. (accession number GQ923958.1, 99.25% identity match). We further confirm whether these isolates matched genera descriptions (Domsch, Gams, & Anderson, 2007). Fusarium, Gibberella, and Microcodicum are mostly known for harboring pathogenic species. In almost all cases, they cause the death of plant tissue (root necrosis or damping‐off diseases) or plant wilts (Leslie & Summerell, 2006; Singleton, Mihail, & Rush, 1992; Smiley, Dernoeden, & Clarke, 2005). Moreover, in grasses, some diseases are known to be caused by co‐infection of species in these three genera (disease complexes) (Xu & Nicholson, 2009). These fungal strains were characterized in terms of colony morphology, conidia type, and intraradical growth by in vitro inoculation on *F. brevipila* seedlings (Fig. S1). Colony and conidia characteristics were measured in pure culture on MEA. Such characterization was necessary for handling these strains during the experiment (e.g., to identify contamination during the inoculum production or during re‐isolations at the end of the experiment).

In vitro tests were performed by aseptically germinating and growing *F. brevipila* in Murashige–Skoog agar (a medium that contains inorganic salts to sustain plant growth, but without any carbon compound). Following this, the seedlings were inoculated with the selected fungi by placing an agar plug of the fungus in direct contact with the root systems (Fig. S2). The inoculated plants were allowed to grow for three more weeks. Then, plants were harvested, dry weight was measured, and the root systems were screened under the stereoscope to identify root necrosis (root lesions) and stained with trypan blue to check fungal infection. These tests showed that all isolates infected the roots, none were pathogenic while their effects on the growth of *F. brevipila* under these conditions were marginal (Fig. S3).

### 2.3 Inoculum production

A single fungal colony growing on potato dextrose agar (PDA) Petri dishes corresponding to the chosen strains were used as the source for the production of inoculum for the experiment. We used the method of inoculum production described in Singleton et al. (1992). This protocol allows production of large amount of solid inoculum that it is easy to handle. The fungi are grown together with an organic substrate, which is then added to soil, reflecting the saprotrophic capabilities of this set of fungi which represent a natural way of infection (Thrall, Bever, Mihail, & Alexander, 1997). Briefly, we used oat kernels as the organic matter substrate. They were soaked in water overnight, autoclaved twice, and inoculated with agar plugs (PDA) from 2‐week‐old colonies. The fungi were grown in the kernels for one month in 500‐ml Erlenmeyer flasks sealed with aluminum foil and parafilm. Gas exchange was allowed by frequently opening the flasks under a sterile hood to avoid contamination. Two erlenmeyers were mock inoculated with sterile agar plugs (PDA) and kept under the same conditions as the fungal inoculum. This material was used to establish a control treatment for the experiment. Some kernels were plated on PDA prior to soil inoculation to verify the lack of contamination in the inocula and mock inoculated control.

### 2.4 Experimental microcosms and experimental design

The experimental microcosms consisted of *F. brevipila* and two other commonly co‐occurring plant species in the Mallnow grassland grown together in 3‐L cylindrical pots (15 cm diameter; 17 cm depth) filled with field collected soil. These experimental microcosms were used in a factorial design with two treatments: a fungal treatment and a soil type treatment. The fungal treatment had five levels: single species inoculations of each of the three fungal isolates (*Fusarium*,* Gibberella*,* Microdochium*, referred as monocultures), a mixture of the three fungal strains, and a mock inoculated control (autoclaved kernels that were never inoculated with the fungi and which remained free of other fungal contaminants). The soil type treatment consisted of two levels: a “high sand” and “low sand” soil type. Each treatment combination was replicated 10 times (five fungal treatments levels × two soil type treatment levels × 10 replicates = 100 microcosms). In the following, we describe how these experimental microcosms and experimental design were assembled.

#### 2.4.1 Plant species

The plants in the microcosms were *A. elatius* (Poaceae), *F. brevipila* (Poaceae) (where the fungi were originally isolated), and *A. elongata* (Plumbaginaceae) (as mentioned above, all three plants were found to commonly co‐occur in our 15 × 15 m plot in the Mallnow Grassland). Seeds of these plant species were collected from wild plants in the Mallnow grassland. The seeds were surface sterilized with NaOCl (5%) and ethanol (70%) and germinated on sterile glass beads, to reduce the chance of contamination via seed surface fungi. Two‐week‐old seedlings were then transferred to the 3‐L gardening pots. In total, two individuals per species were used in each microcosm, giving a total density of six plant individuals per microcosm. The plants were spaced 3 cm from the border of the pot and 3 cm from each other.

#### 2.4.2 Soil treatment

The soil used in the experiment was collected from two sites at the Mallnow grassland (to a maximum depth of 30 cm), approximately 50 m away from the 15 × 15 m plots from which the fungi were isolated. At one site, the soil consisted mostly of sand without vegetation cover, while the other site was covered by vegetation similar to the one observed in the 15 × 15 m plot. Both soils were sieved (3 mm mesh size) to remove large pieces of material. The “high sand soil type” was prepared using a 1:1 mixture of field collected soil and sand, while the low sand soil type was prepared using directly field collected soil from the site that had vegetation cover. The soils were then steam sterilized twice for 4 hr at 100°C (48 hr in between steaming sessions) to eliminate other soilborne fungi (samples of the soil after steaming were plated on PDA to confirm the elimination of fungi). A total of 500 grams soil samples of each type were taken and sent for chemical analysis to the LMS Lufa Rostock Laboratory (Rostock, Germany). Results from this analysis are summarized in Table 1.

**Table 1:** Chemical analysis of the two soil types used in this study

Soil type	CaCl_2_	Double lactate extraction			CaCl_2_ extraction		CaCl_2_/DTPA extraction			
	pH	P_2_O_4_, mg/100 g	K, mg/100 g	Mg, mg/100 g	NO_3_, mg/100 g	NH_4_, mg/100 g	B, mg/Kg	Cu, mg/Kg	Mn, mg/Kg	Zn, mg/Kg
High sand	7.1	1.5	2.0	9.0	1.3	0.2	0.18	1.2	15	1.1
Low sand	7.3	4.9	2.0	18.0	1.5	0.1	0.06	0.5	9.0	1.0

#### 2.4.3 Fungal treatment

To create the fungal treatments, we used a soil infestation protocol (Singleton et al., 1992): inoculum of each fungal species and the mock inoculated kernels for the control was added and mixed to the soil before transplanting the plants (soil infestation). We applied 100 ml of each inoculum to the 3‐L pots (1% of the volume of the pot). For the mixture treatment, we applied and mixed 33 ml of the inoculum of each fungal species (keeping constant the total amount of inoculum as in the single inoculation treatments and control).

#### 2.4.4 Glasshouse conditions

All microcosms were maintained from January to April 2012 in our fully climate‐controlled glasshouse facility at the FU‐Berlin (12 hr of artificial light by Phillips Son‐T Agro lamps, 400 W, 16,000 Lumen; 20°C/18°C temperature day/night; 45.5% relative humidity). Plants were watered twice a day with 15 ml (first month), 30 ml (second month), and 60 ml of tap water until the end of the experiment (water regime was changed to meet the increase in growth of the plants; given the size and amount of experimental units, we were not able to use autoclaved sterile water but softened water). No additional nutrients were given during the experiment.

### 2.5 Harvest

We harvested the two grasses 3 months in the experiment to reduce their shading on *A. elongata* (grasses were clipped 3 cm above the soil). The second and final harvest was performed 1 month after the first. This harvest was performed by clipping all three species at ground level. The material of both harvest was placed in a drying oven at 50°C for 1 month, and dry weight was measured. Dry weight was used as our measurement of aboveground productivity of the entire microcosm and each individual species. We used this productivity as a proxy of the fitness of each species. We consider this an appropriate proxy for our experiment because all plants were in vegetative phase (no flowering). The use of this variable as proxy of fitness is a common practice for short‐term greenhouse studies aiming at detecting outcome of plant–plant competition (Gibson, Connolly, Hartnett, & Weidenhamer, 1999) and plant–fungal interactions (Wagg, Jansa, Schmid, & van der Heijden, 2011) (the aims of our study).

The whole root system of each microcosm was destructively harvested without distinguishing plant species, washed with tap water to remove soil, oven dried, and weighed. In half of the replicates per treatment (50 pots), we took subsamples of the root system prior drying for microscopy of infection and re‐isolation of the inoculated fungi. We took sixty root segments (1 cm) at different areas of the root system (i.e., the root segments were not taken from the same area of the root system, increasing the likelihood of include root segments from all plant species), as it was not possible to separate root systems per plant species. The root segments were surface sterilized using the aforementioned protocol and placed on MEA. The number of retrieved colonies per fungal isolates was recorded. Determinations of inoculated fungi and contaminants were easy to assess given characteristic features in colony morphology of the isolates when grown in pure culture in MEA at 25°C (although our greenhouse is completely climate‐controlled, conditions are not fully sterile. Contamination may happen through airborne propagules). Moreover, the morphology of the retrieved colonies matched the characteristics of the fungi before the experiment, and an additional five colonies per isolate taken from different microcosms were transferred to individual plates and were RFLP profiled. In addition, we collected 20 cm root systems in three pots per treatment combination, which were assessed for presence of fungal intraradical structures by trypan blue staining and light microscopy (Phillips & Hayman, 1970). Colonization was measured as the number of hyphal structures found in 100 views at 40× magnification.

The *Fusarium* strain produced fast growing colonies, with few aerial (flat) white mycelia and a filamentous margin. *Gibberella sp* produced very distinctive dome‐shaped mycelia with orange color from above and scarlet from below, within a week colonies became radially striate with a lobate edge. *Microdochium* sp produced very flat mycelium with an undulate margin; colonies initially were salmon colored from above but became olivaceous black as chlamydospores were produced after a week of growth (Fig. S4).

### 2.6 Data analysis

We used colonization and re‐isolation (presence/absence) data to assess the presence of the fungi at the end of the experiment. Data were analyzed as counts (number of retrieved colonies, number of observed fungal structures) using generalized linear models, with quasi‐poisson error structure to correct for overdispersion.

We analyzed aboveground plant productivity using two‐way ANOVA, where the fungal treatment (with five levels: a control, three fungal monocultures, and a fungal mixture) and soil type (with two levels: low and high sand) were used as factors. This analysis was performed to determine how fungal endophyte community composition modified the way plants responded to soil type levels (hypothesis two) which is indicated by the soil type × fungal treatment interaction term. Then, we conducted separate one‐way ANOVA, followed by pot hoc Tukey HSD test, on each soil type, to test whether: (1) the fungal monocultures had significant effects in plant productivity, by comparing to their respective controls and whether the fungal monocultures had species‐specific effects, by comparing monocultures among each other (hypothesis one); (2) the effect of the monocultures is affected by the presence of other fungi, by comparing to the fungal mixture (hypothesis three).

We used two‐way MANOVA to test for the effects of fungal treatment, soil type, and their interaction on plant community structure. Effects on community structure are the product of differential fitness among plant species in the community. Thus, we use the shoot biomass of each plant species as our measure of plant fitness, as it is commonly used in experimental microcosms’ communities (Wagg et al., 2011). For significant effects detected by MANOVA, we proceeded with one‐way ANOVA on the biomass of each plant species followed by post hoc Tukey HSD pair wise comparisons. As with overall plant productivity, this analysis was performed to test whether (1) the fungal monocultures had significant effects on the productivity of each plant species, by comparing them to respective controls; (2) the fungal monocultures had species‐specific effects on each species, by comparing monocultures among each other; (3) the effect of the monocultures is affected by the presence of other fungi, by comparing them to the respective fungal mixture.

Additionally, we tested whether the changes in relative biomass caused by the fungi altered competitive interactions by creating a correlation matrix of the biomass of the three interacting species for each soil × fungal treatment combination. Negative correlations indicate competitive interactions (Goldberg & Landa, 1991). All the analyses were performed in R (R Development Core Team, 2011).

We used nontransformed data of aboveground productivity (total biomass of the three plant species) as there were no significant deviations from assumptions of normality of residuals and homoscedasticity of variances after removal of outliers (2 microcosms that were water‐logged and one microcosm in the *Microdochium* low sand treatment where the biomass was 2.1 times larger than the interquartile range above the third quartile of that group (Crawley, 2012)).

In the case of the biomass of the two grasses, we used nontransformed data of the biomass as there were no significant deviations from normality of residuals or homoscedasticity of variances. In the case of *A. elongata*, Bartlett test detected heteroscedasticity and no transformation solved the issue. However, visual inspection of the error distribution indicated that the heteroscedasticity was due to three values in three different treatments while no correlation between the mean and error was observed. Therefore, we also used nontransformed data of *A. elongata* in the analysis.

## 3 Results

### 3.1 Re‐isolations and microscopy

Re‐isolation data showed that the control treatment remained contamination free while there was successful recovery of *Fusarium* and *Gibberella* (Fig. S5) from treatments where they were used as inoculum. Although it was not possible to re‐isolate *Microdochium*, fungal structures (hyphae, spores) were commonly observed (Fig. S4). Furthermore, in this treatment, typical *Microdochium* chlamydospores were frequently observed in the cortex and root hairs (such spores are also observed in pure *Microdochium* cultures on MEA and during the in vitro test on *F. brevipila* (Fig. S4). Such chlamydospores are not produced by any other of the isolates used, and it is very unlikely that contaminants could produce such features. Moreover, this type of chlamydospore has been frequently reported during colonization of endophytic *Microdochium* strains in temperate grasses (Mandyam et al., 2010). Further, we did not identify any AMF structures in the roots, indicating that these fungi were absent from the soil and did not drive any of the plant growth effects.

### 3.2 Effects on plant aboveground productivity

Overall, soil type, fungal treatment, and their interaction significantly altered aboveground productivity in the microcosms (Table 2). The significant interaction term indicates that plants responses to fungal treatments were dependent on soil type. Productivity was lower in the high sand soil type than in the low sand soil type and separate ANOVAs on each soil type showed that plant productivity was significantly affected by fungal treatment (monocultures and mixtures) (high sand: *F = *8.60, *p < *.001; low sand: *F *=* *6.82, *p *<* *.001).

**Table 2:** Two‐way ANOVA results for total productivity (aboveground and belowground) and for each individual species using soil texture and fungal identity as factors. Analysis based on nontransformed data. Statistical significance levels of F. ratio test are indicated as: ***p<0.001, **p<0.01, *p<0.05

Source of variation	*df*	*MS*	*F*‐ratio
Total aboveground productivity			
Soil texture	1	125.45	48.70***
Fungal identity	4	28.86	11.20***
Soil texture × fungal identity	4	10.94	4.25**
Residuals	86	2.58	
Total belowground productivity			
Soil texture	1	109.58	19.74***
Fungal identity	4	39.02	7.03***
Soil texture × fungal identity	4	3.73	0.67
Residuals	86	5.55	
*Arrhenatherum elatius* biomass			
Soil texture	1	132.12	47.89***
Fungal identity	4	25.68	9.31***
Soil texture × fungal identity	4	8.71	3.16*
Residuals	86	2.76	
*Festuca brevipila* biomass			
Soil texture	1	0.0005	0.0022
Fungal identity	4	0.52	2.21
Soil texture × fungal identity	4	0.61	2.62*
Residuals	86	0.23	
*Armeria elongata* biomass			
Soil texture	1	0.07	3.70
Fungal identity	4	0.11	5.70***
Soil texture × fungal identity	4	0.05	2.57*
Residuals	86	0.01	

In the high sand treatment, aboveground plant productivity showed little variation in the *Fusarium* and *Gibberella* monocultures compared to the control, but in the *Microdochium* monoculture and in the mixture there was reduction of 11% and 13% in aboveground plant productivity, respectively (although only the latter reduction was significant *p* = .03 Tukey HSD test). The effect of the mixture was significantly different to the *Fusarium* and *Gibberella* monocultures (*p* < .001 in both cases, Tukey HSD test), but not to *Microdochium* one (*p* = .99, Tukey HSD test) and the same pattern holds for belowground productivity.

**Figure 1:**
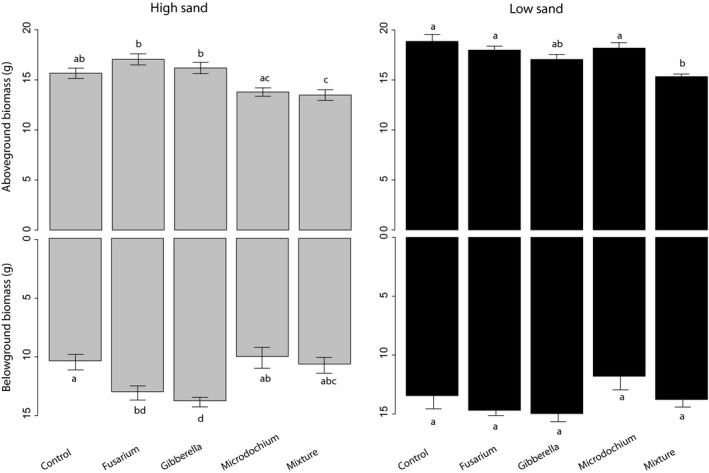
Total productivity of microcosms in response to soil texture and fungal identity. Gray bars indicate high sand (low fertility) treatment (left); black bars indicate low sand (high fertility treatment (right). Bars indicate mean values and standard errors, and different letters indicate significant differences among treatments

In the low sand treatment, aboveground productivity responses to fungal monocultures were neutral in all cases (no significant differences compared to the control) and variation among monocultures was also not significant. However, the plant productivity in the fungal mixture was significantly reduced (18% reduction compared to the control) and it was also significantly different to the monocultures (except for *Gibberella*). Aboveground productivity in the mock inoculated control, monocultures, and mixtures for each soil type are summarized in Fig. 1.

### 3.3 Effects on plant community structure

**Figure 2:**
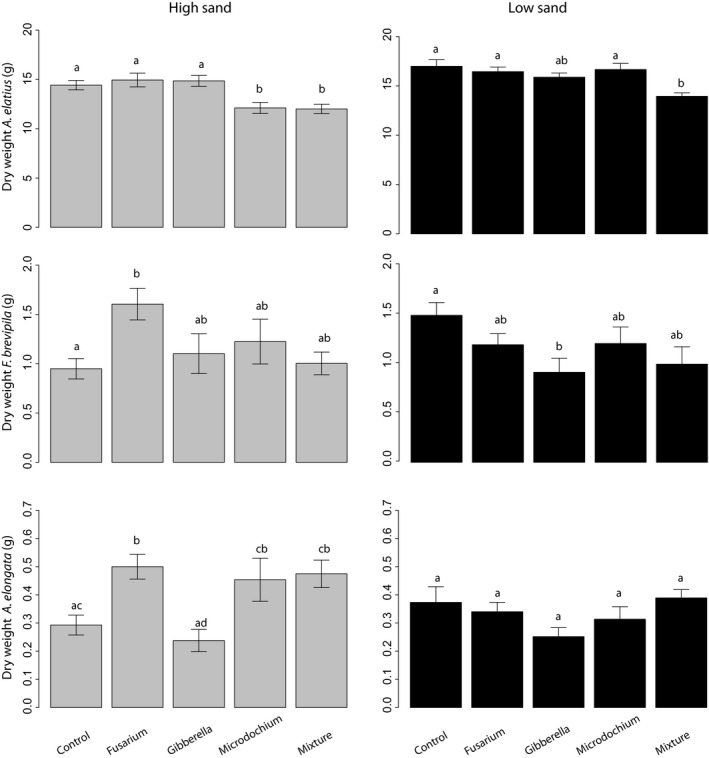
Biomass responses to soil texture and fungal identity of the three species used in the experiment: top, *Arrhenatherum elatius*; middle, *Festuca brevipila*; bottom *Armeria elongata*. Gray bars indicate high sand (low fertility) treatment (left); black bars indicate low sand (high fertility treatment (right). Bars indicate mean values and standard errors, and different letters indicate significant differences among treatments

The plant community structure in our microcosms differed significantly depending on soil type, fungal treatment, and the interaction of the two factors, as indicated by the two‐way MANOVA (Table 3). Overall, all plants produced more shoot biomass in the low sand treatment compared to the high sand treatment. However, shoot biomass strongly depended on the fungal soil treatment combination. This, together with the significant soil type × fungal treatment interaction term, shows that the plant response to the changes in soil type was dependent on the fungal endophyte treatment. Aboveground productivity of each plant species is summarized in Fig. 2.

**Table 3:** Two‐way MANOVA results for community structure (changes in biomass of the three plant species used in the experiment) having soil textures and fungal Identity as factors. Analysis based on nontransformed data. Statistical significance levels of F. ratio test are indicated as: ***p<0.001, **p<0.01, *p<0.05

Source of variation	*Df*	Pillai trace	*F*
Soil texture	1	0.40	19.06***
Fungal identity	4	0.60	5.42***
Soil texture × fungal identity	4	0.34	2.78**
Residuals	86		

The largest plant was *A. elatius* in terms biomass produced. In the high sand treatment, the response of this plant to fungal monocultures was neutral for *Fusarium* and *Gibberella*, but its shoot biomass was significantly reduced in the *Microdochium* monoculture and in the fungal mixture (16% less shoot biomass compared to the control in both cases, *p* < .05, Tukey HSD test). The effect of the fungal mixture was significantly different to the *Fusarium* and *Gibberella* monocultures but not to the *Microdochium* one. In the case of *F. brevipila*, the shoot biomass in the fungal monoculture of *Fusarium* had a large positive effect compared to the control (plants of this species produced 70% more biomass compared to the control, *p* < .05, Tukey HSD test), while the other fungal monocultures and the mixture did not differ from the control. Despite this positive effect compared to the control, shoot biomass of *F. brevipila* in the *Fusarium* monoculture was not significantly different from the other two monocultures. The shoot biomass of this plant in the fungal mixture did not differ significantly from any of the fungal monocultures, but it was marginally significant to the *Fusarium* monoculture (*p* = .09) (plants were on average almost 60% smaller in the mixture treatment compared to the *Fusarium* monoculture). *Armeria elongata* produced more shoot biomass in the monocultures of *Fusarium* (70% more) and *Microdochium* (54% more) and the in the mixture (62% more) compared to the ones in the control, although it was only significant in the first case (*p* < .05, Tukey HSD test), while plants were neutral to the *Gibberella* monoculture. The effect of the mixture was not significantly different from any of the monocultures, except for *Gibberella*, and it was of a similar magnitude as *Fusarium* and *Microdochium*.

Correlation coefficients among the interacting plants in this soil treatment (Table S1) showed that there was a significant negative correlation between the biomass of the two grasses in the *Fusarium* and *Microdochium* treatments (*Fusarium*,* r* = .78, *p* < .05; *Microdochium*,* r* = −.66, *p* < .05) while there was no correlation observed in the control suggesting that the presence of *Fusarium* and *Microdochium* increased competitive interactions between the grasses.

In the low sand treatment, the shoot biomass of *A. elatius* in the fungal monocultures was not significantly different from the control, but in the fungal mixture plants produced 17% less biomass compared to the control. Shoot biomass of this plant did not significantly vary across monocultures. However, the biomass in the mixture was significantly different from the monocultures, except to *Gibberella*. The biomass response of *F. brevipila* to the fungal treatments was weak. None of the fungal treatments (monocultures and mixture) were significantly different to the control except in the *Gibberella* monoculture, where plants produced 18% less biomass. However, the biomass of this plant in this monoculture was not statistically different from the other monoculture nor to the mixture. This weak response to fungal treatments also holds for *A. elongata*. None of the fungal treatments (monocultures and mixture) were significantly different from the control. The monocultures did not differ significantly from each other nor to the mixture (Fig. 2).

## 4 Discussion

We provide evidence that changes in REF species can cause differential host growth responses among plants in our experimental communities. Furthermore, such effects greatly modified the way plants respond to the differences in soil types imposed in this study. For example, just due to the presence of our *Fusarium* strain, both *F. brevipila* and *A. elongata* produced more shoot biomass in the high sand treatment compared to the plants grown in the control low sand treatment (which had higher nutrient concentration compared to the high sand treatment, see Table 1), despite the presence of the fast growing plant *A. elatius*. In the dry grassland to which our experiment relates, our soil types reflect the strong differences in soil parameters that affect host growth (texture, pH, and nutrient content), which are well‐known abiotic parameters to drive changes in plant community structure. It remains to be tested how those soil parameters modify the community structure of REF in the grassland and its feedback on plant communities.

Many possible mechanisms could explain the plant growth responses in this experiment. Reduction in biomass production in response to root endophytic colonization can be seen as weak parasitism (Mandyam, Roe, & Jumpponen, 2013), or as induction of host resistance resulting in the allocation of carbon to the production of expensive defense compounds rather than to vegetative growth (Aimé, Alabouvette, Steinberg, & Olivain, 2013; Heil & Baldwin, 2002). Positive effects could be due to production of plant growth hormones (Schulz & Boyle, 2005), or transport of nutrients to the host as a result of mineralization of organic matter (given the saprotrophic capabilities of these fungi) (Newsham, 2011). Clearly, information about the symbiotic and saprotrophic traits of the used strains is needed to disentangle these mechanisms, but is beyond the scope of the present study.

The consequences of these differential plant growth responses to REF may result in interesting cases of indirect ecological interactions. For example, when some plant species suppress the growth of competing neighbors by hosting fungal species detrimental to others, this has been referred to as “apparent competition” (Beckstead, Meyer, Connolly, Huck, & Street, 2010; Hatcher, Dick, & Dunn, 2006). Indeed, previous studies have reported soilborne fungi as drivers of this sort of indirect interactions (Holah & Alexander, 1999; Van der Putten & Peters, 1997). In this experiment, apparent competition may explain the negative relationship between the yields of the two grasses *A. elatius* and *F. brevipila* in the presence of *Fusarium* and *Microdochium* in the high sand treatment, and in the mixture in the low sand treatment combination.

Furthermore, it is evident that the exact nature of the host response, the mechanism driving it, and its outcome on host communities greatly depends on soil conditions. For example, the large positive responses of *F. brevipila* and *A. elongata* to *Fusarium* were only observed in the high sand treatment (plants doubled their shoot biomass compared to the respective control), while in the low sand treatment differences with respect to the control were small and nonsignificant. This effect may be due to fungal mineralization (saprotrophic) capabilities which is known to depend on availability of inorganic versus organic nutrient sources (Mayerhofer, Kernaghan, & Harper, 2013). That is, REF could have made available nutrients present in the soil organic matter. At the high sand treatment level of the present study, the organic material added to soil in the inoculum (oat kernels) could have constituted a pool of nutrients that was only available when fungi were present. As organic matter is added to the soil as litter under natural settings, the saprotrophic capabilities of these fungi may play an important role for plant growth. Thus, we consider that determining the extent to which saprotrophic capabilities among REF are responsible for plant growth promotion effects represents an important question in plant‐endophyte research. Regardless of the exact mechanisms, in our experiment, the presence of the fungi was able to reduce host fitness differences of the interacting plant species (they behave as “equalizing” factors (Chesson, 2000)) and thus promote diversity maintenance.

Our experimental manipulations (monocultures vs. mixtures) also show that endophyte–endophyte interactions have important implications on host community structure. For example, in the high sand treatment, the effects seen in the mixture treatment were similar to the ones observed in one of the monocultures suggesting that one fungus dominates under these conditions (reduction in biomass on *A. elatius* as in the Microdochium monoculture); while in the low sand treatment, the effects seen in the mixture treatment does not correspond to any the effects seen in the monocultures, despite the presence of all fungi in the mixture treatments, indicating that fungal–fungal interactions played a role. These results are congruent with similar studies on AMF. Wagg et al. (2011) also showed that competitive dominance and complementarity among three AMF species that were present together in the soil of experimental microcosms depended on soil type. Further experiment is required to determine the exact nature of the interactions (e.g., competition) and their consequences for fungal community structure (fungal abundance patterns). Our inoculation scheme created a situation where a single strain could occupy most of the rhizosphere of three hosts without any other fungal competitors. This is an unlikely scenario in natural systems, but using this information future studies can add increasing complexity to measure how other interactions (e.g., AMF, rhizosphere bacteria) influence the effects reported in this study (it would be interesting, e.g., to determine the extent to which soil and rhizosphere bacteria present in our study explain the responses of the plants to REF species). Rillig et al. (2014) used this approach, showing that both AMF and REF communities alone did not have any effect on plant growth promotion when inoculated in isolation in experimental microcosms, but they reduced plant growth when co‐inoculated.

Because our experimental microcosms mimicked an actual community module (Hatcher et al., 2006) (both fungi and plants do co‐occur within a 15 × 15 m plot in a natural grassland), it is likely that the responses observed in this experiment play an important role in nature. Thus, this experiment adds phenomenological evidence to our understanding of how the REF species may drive plant community structure by their direct influence on plant growth as well as indirectly by affecting the way plants respond to soil abiotic factors. Further studies should aim at evaluating long‐term effects of manipulations of REF composition, structure, and diversity on plant community dynamics.

## Conflict of Interest

None declared.

## Funding Information

German Academic Exchange Service.
