# Supplementary material for: Effect of different root endophytic fungi on plant community structure in experimental microcosms
Source: Ecol Evol. 2016 Oct 18;6(22):8149–58. doi: 10.1002/ece3.2416 (PMC5108266; doi:10.1002/ece3.2416)
Supplement: Supplementary file 1 [file ECE3-6-8149-s001.docx]

**Electronic Supplementary Material (ESM)**

**Effect of different root endophytic fungi on plant community structure in experimental microcosms**

**Journal: Ecology and Evolution**

Carlos A. Aguilar-Trigueros^1,2*^, Matthias C. Rillig^1,2^

^1^Freie Universität Berlin, Institut für Biologie, Plant Ecology, D-14195 Berlin, Germany

^2^Berlin-Brandenburg Institute of Advanced Biodiversity Research, D-14195 Berlin, Germany

*Correspondence to Carlos A. Aguilar-Trigueros

Freie Universität Berlin, Institut für Biologie, Plant Ecology, Altensteinstr. 6, D-14195 Berlin, Germany; Email: calgit@gmail.com, Tel +49 30 838-53143


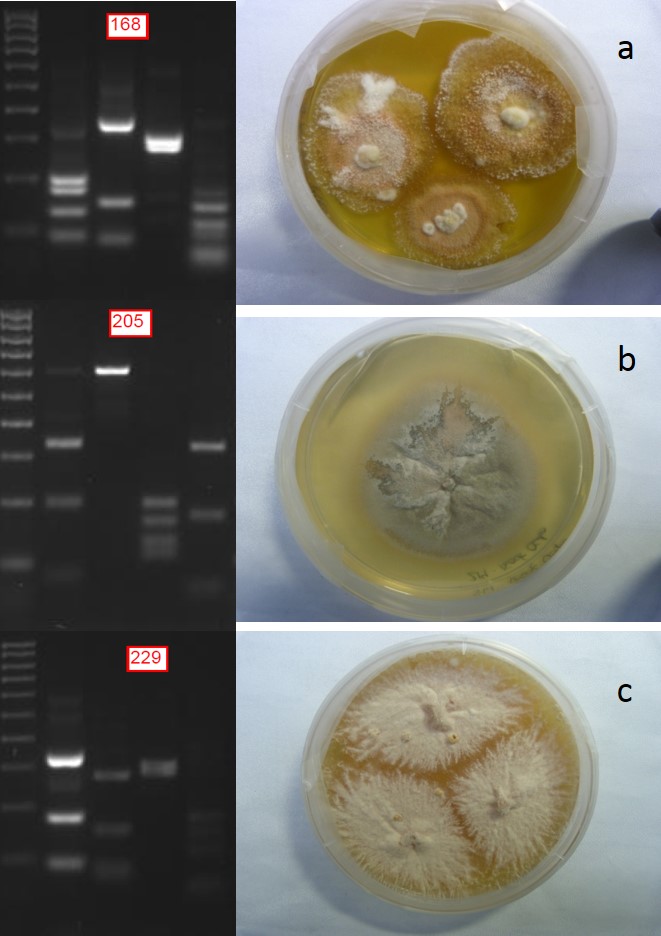


Fig. S1. RFLP patterns and matching colony morphology types. a) Isolate 168 corresponding to *Gibberella sp;* b) Isolate 205, *Microdochium sp*; c) Isolate 229, *Fusarium sp*.


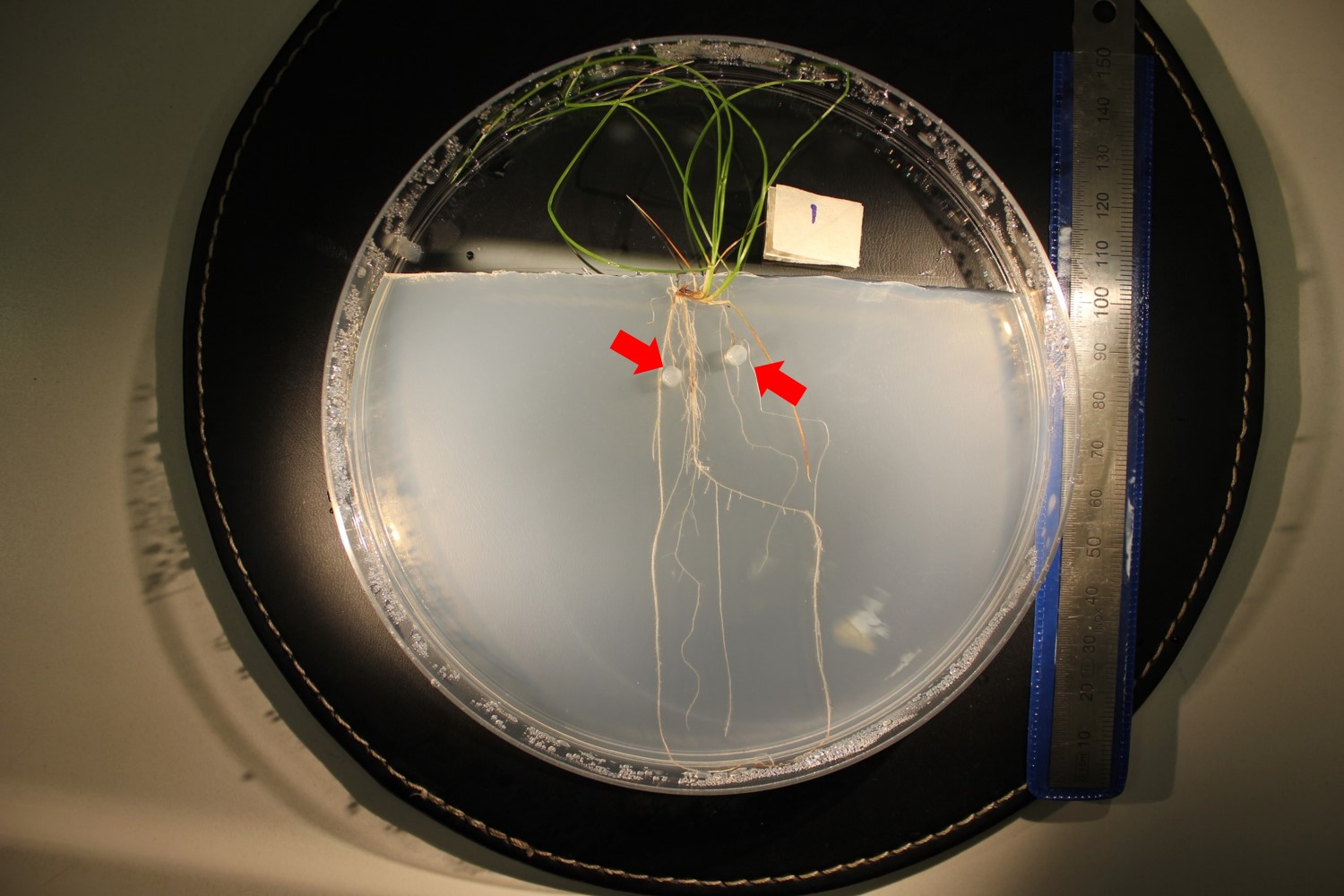


Fig. S2. Picture of the experimental set up for the in vitro test of the selected fungi on *F. brevipila seedlings.* Red arrow indicate the agar plugs with fungal mycelia at the moment of inoculation. Plates were kept in vertical position during the experiment.


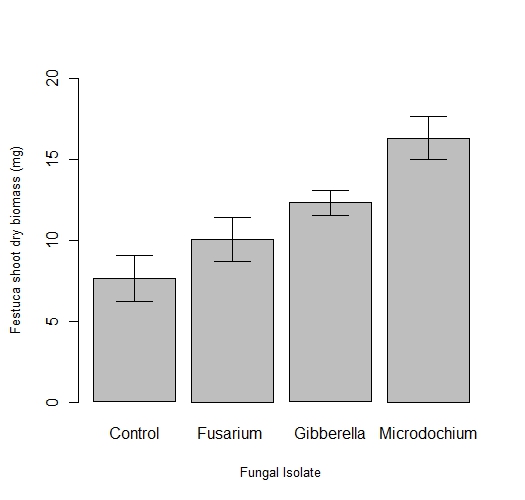


Fig. S3. Responses of *Festuca brevipila* to *in vitro* inoculations with the three fungal isolates used in the main experiment. Responses obtained from two week old seedlings that were inoculated with each isolates and let them grow together for growing 6 weeks (Sample size: Control: 8 seedlings; Fusarium: 5 seedlings, Gibberella: 5 seedlings, Microdochium 6 seedlings) . Bars indicate means, whiskers standard errors.


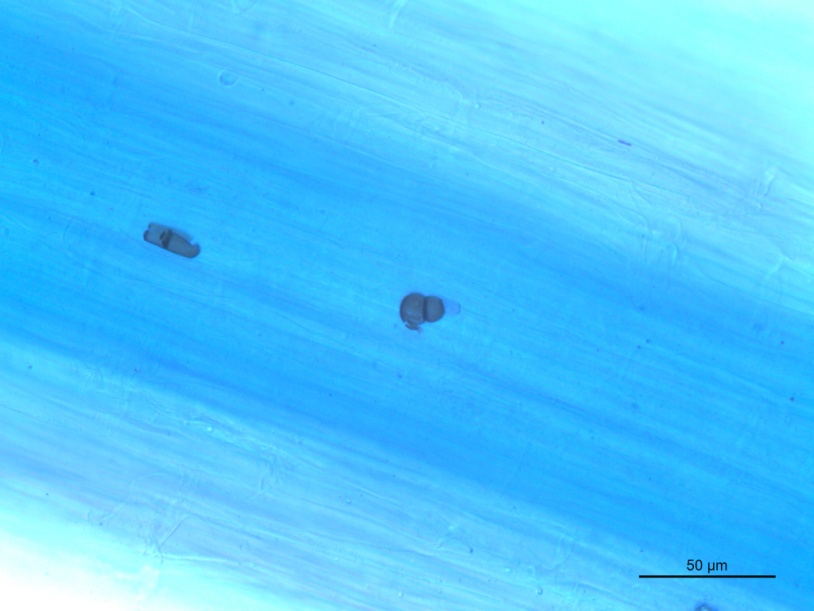

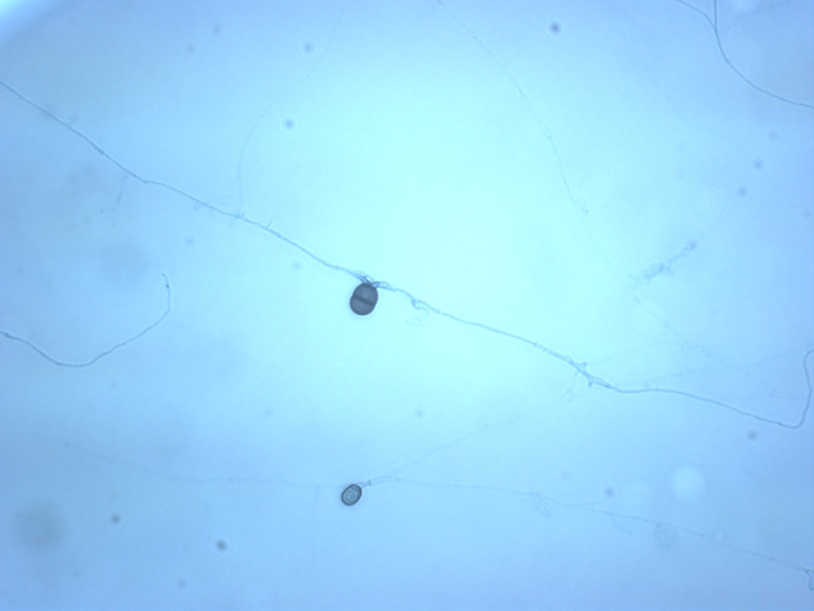

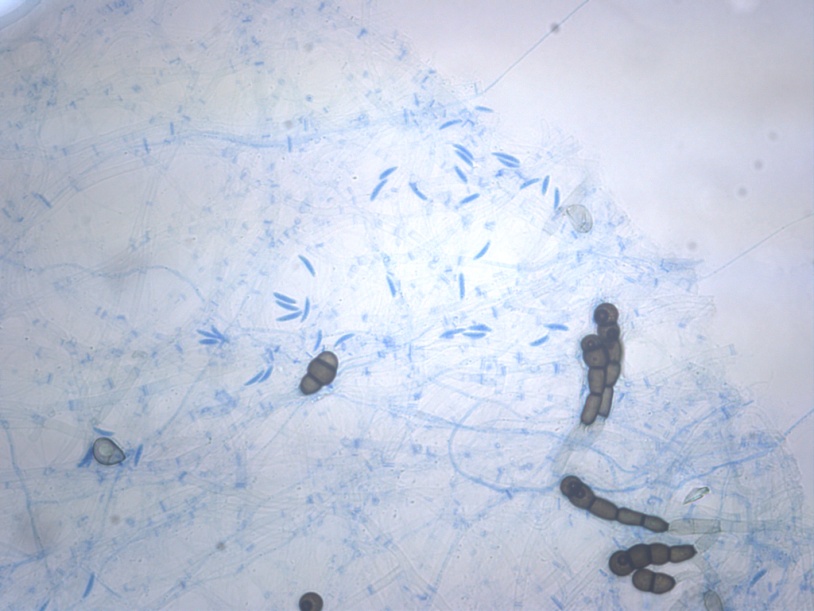


a

d

c

b


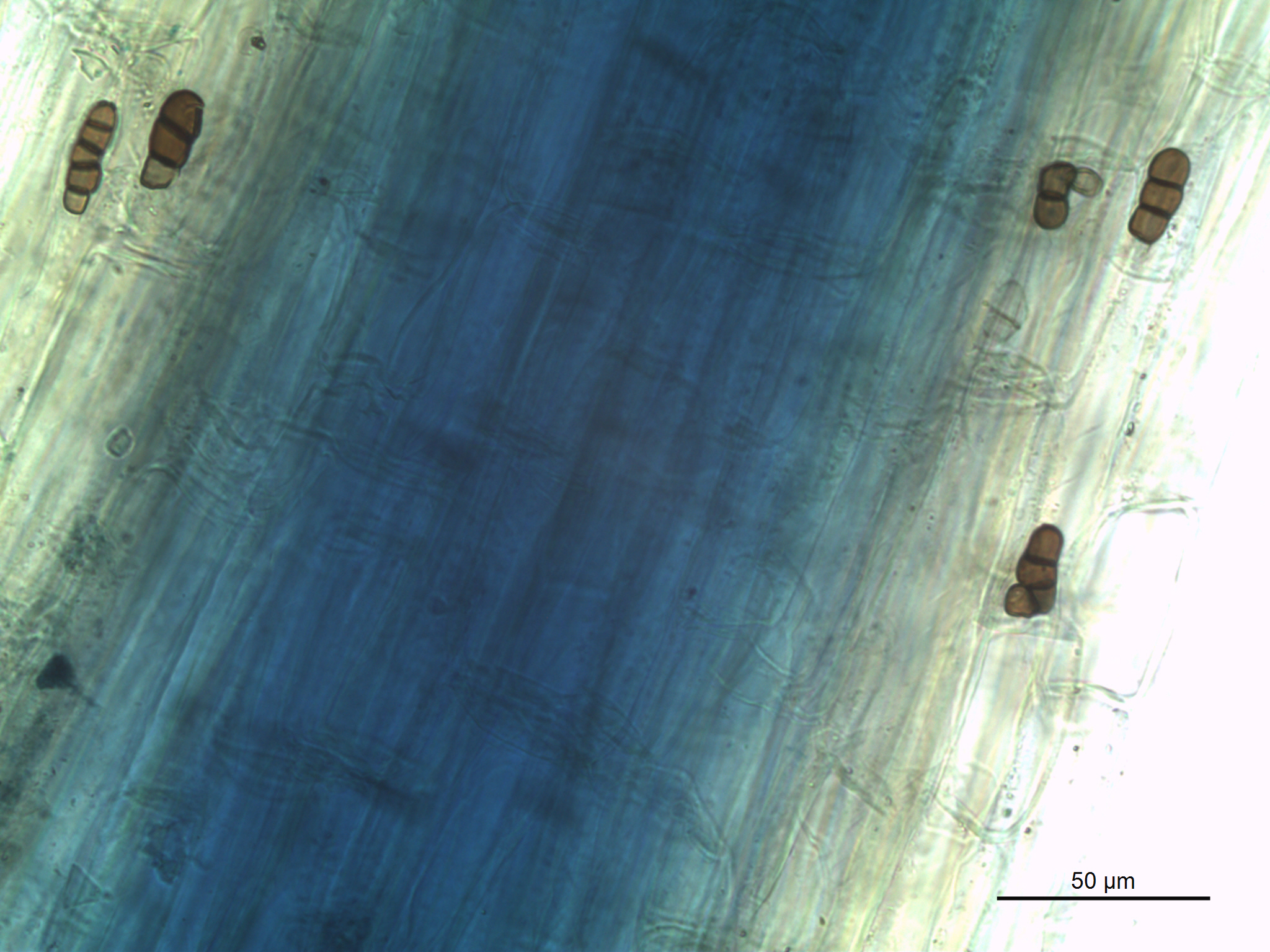


b

Fig. S4. Comparison of chlamydospores of *Microdochium* isolates in pure culture (c and d) with observed ones in *Microdochium* treatments growing in the roots of *F. brevipilla* (a and b)*.* In b, the chlamydospores are inside the root cortical cells (black arrow), where they were frequently observed. In d) the *Microdochium* isolates also produce microconidia (red arrow).


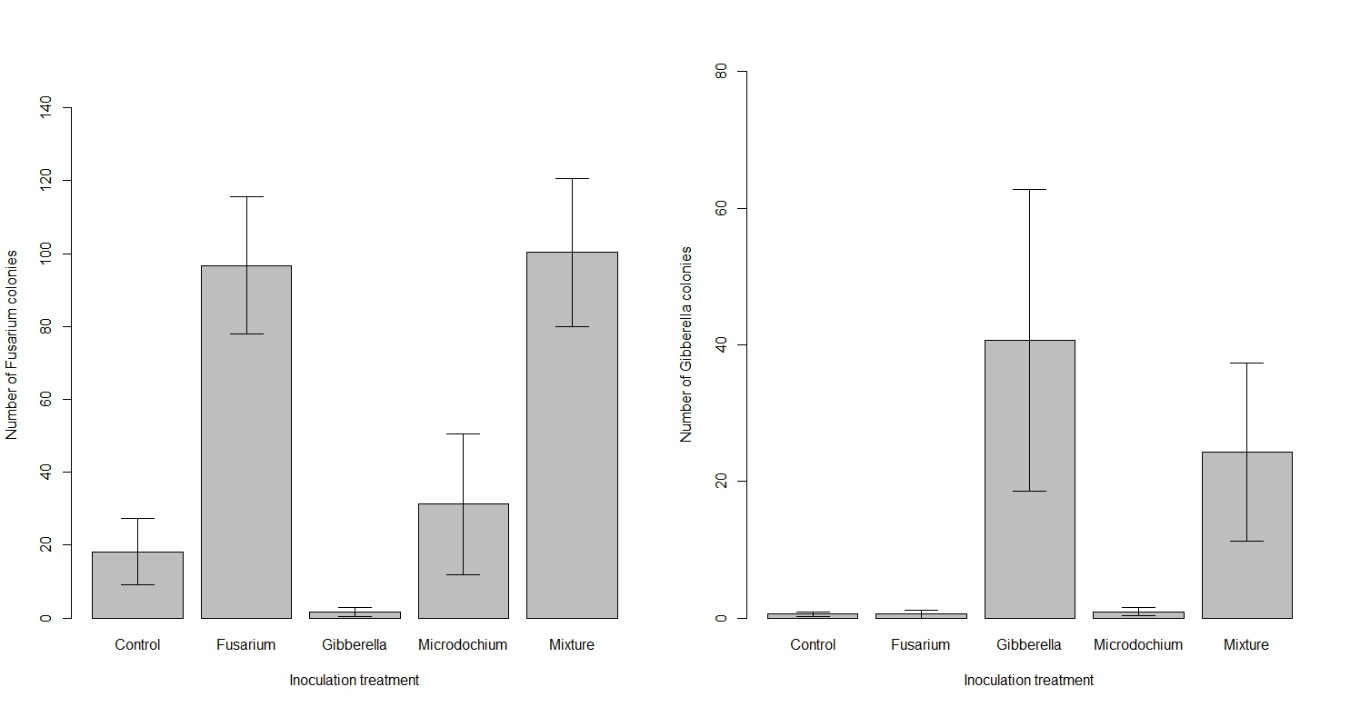


Fig. S5. Fungal re-isolations from microcosms at the end of the experiment. The *y-*axis corresponds to the total number of fungal colonies of *Fusarium* (left barplots) and *Gibberella* (right barplots) retrieved from 10 pots from each fungal treatment (5 in Low Sand and 5 in High Sand). Barplots represent means and the whiskers standard errors.


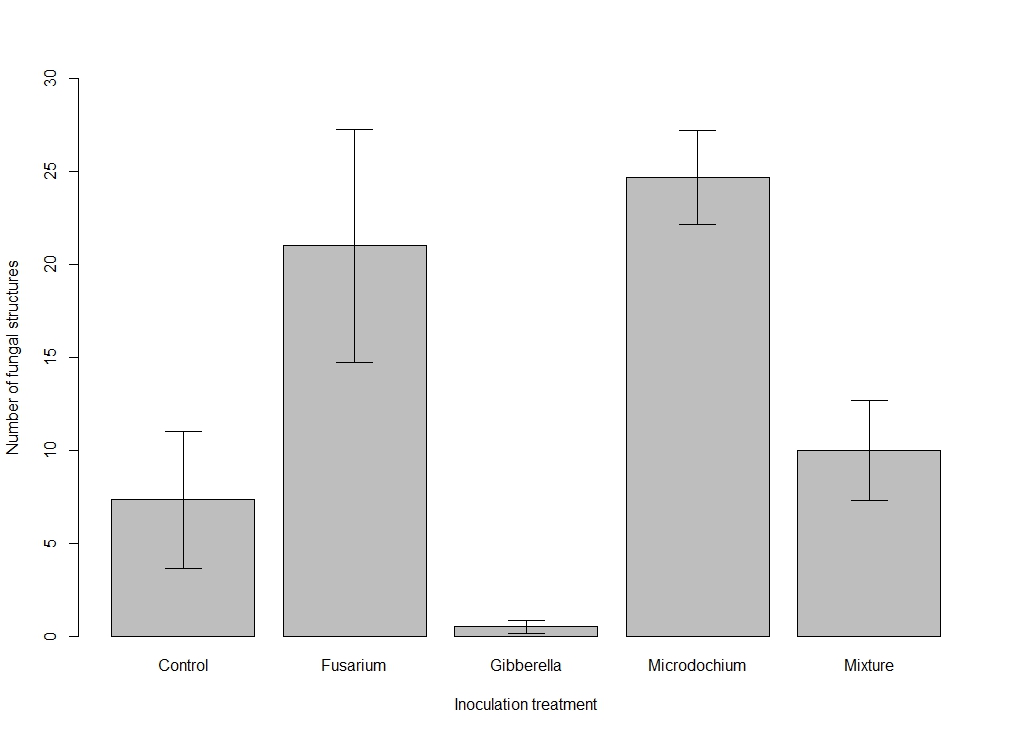


Fig. S6. Number (mean and standard error) of fungal structures found in the roots of in the microcosm. *y* axis correspond to number of fungal structures that were detected up to 100 views. The sample consisted of 20 cm of root system in three microcosms per treatment combination Barplots indicate means and whiskers, standards errors.

Supplementary table

Table S1. Correlation matrix of the biomass of each interacting species in each fungal treatment within soil types. Values indicate correlation coefficients (r), asterisks indicate statistical significance (p<0.05).

| High Sand soil type | | | |  | Low Sand soil type | | | |
| --- | --- | --- | --- | --- | --- | --- | --- | --- |
| CONTROL HS | **Arrhenatherum** | **Festuca** | **Armeria** |  | **CONTROL LS** | **Arrhenatherum** | **Festuca** | **Armeria** |
| Arrhenatherum | 1 | 0.11 | 0.51 |  | **Arrhenatherum** | 1 | 0.03 | 0.2 |
| Festuca |  | 1 | -0.08 |  | **Festuca** |  | 1 | 0.66* |
| Armeria |  |  | 1 |  | **Armeria** |  |  | 1 |
|  |  |  |  |  |  |  |  |  |
| FUSARIUM HS | **Arrhenatherum** | **Festuca** | **Armeria** |  | **FUSARIUM LS** | **Arrhenatherum** | **Festuca** | **Armeria** |
| Arrhenatherum | 1 | -0.78* | -0.56 |  | **Arrhenatherum** | 1 | -0.55 | -0.35 |
| Festuca |  | 1 | 0.22 |  | **Festuca** |  | 1 | 0.31 |
| Armeria |  |  | 1 |  | **Armeria** |  |  | 1 |
|  |  |  |  |  |  |  |  |  |
| GIBBERELLA HS | **Arrhenatherum** | **Festuca** | **Armeria** |  | **GIBBERELLA LS** | **Arrhenatherum** | **Festuca** | **Armeria** |
| Arrhenatherum | 1 | -0.2 | 0.53 |  | **Arrhenatherum** | 1 | 0.26 | 0.85* |
| Festuca |  | 1 | -0.04 |  | **Festuca** |  | 1 | 0.59 |
| Armeria |  |  | 1 |  | **Armeria** |  |  | 1 |
|  |  |  |  |  |  |  |  |  |
| MICRODOCHIUM HS | **Arrhenatherum** | **Festuca** | **Armeria** |  | **MICRODOCHIUM LS** | **Arrhenatherum** | **Festuca** | **Armeria** |
| Arrhenatherum | 1 | -0.66* | -0.48 |  | **Arrhenatherum** | 1 | -0.45 | -0.33 |
| Festuca |  | 1 | 0.37 |  | **Festuca** |  | 1 | 0.15 |
| Armeria |  |  | 1 |  | **Armeria** |  |  | 1 |
|  |  |  |  |  |  |  |  |  |
| MIXTURE HS | **Arrhenatherum** | **Festuca** | **Armeria** |  | **MIXTURE LS** | **Arrhenatherum** | **Festuca** | **Armeria** |
| Arrhenatherum | 1 | 0.18 | 0.15 |  | **Arrhenatherum** | 1 | -0.65* | -0.55 |
| Festuca |  | 1 | 0.33 |  | **Festuca** |  | 1 | 0.56 |
| Armeria |  |  | 1 |  | **Armeria** |  |  | 1 |

Table S2. Summary of the number of isolates and RFLP-types obtained per plant individual during our sampling campaign. On average, we recovered 9.5 isolates and 3.5 RFLP-types per plant individual.

| *Plant-ID* | *Number of Isolates* | *Number of RFLPs* |
| --- | --- | --- |
| F.brevipila-2 | 10 | 3 |
| F.brevipila-5 | 18 | 4 |
| F.brevipila-6 | 13 | 5 |
| F.brevipila-8 | 6 | 3 |
| F.brevipila-10 | 24 | 11 |
| F.brevipila-11 | 5 | 4 |
| F.brevipila-14 | 2 | 1 |
| F.brevipila-16 | 18 | 6 |
| F.brevipila-18 | 13 | 3 |
| F.brevipila-20 | 9 | 5 |
| F.brevipila-21 | 14 | 3 |
| F.brevipila-22 | 8 | 3 |
| F.brevipila-24 | 12 | 5 |
| F.brevipila-27 | 4 | 1 |
| F.brevipila-28 | 6 | 2 |
| F.brevipila-29 | 10 | 1 |
| F.brevipila-31 | 5 | 3 |
| F.brevipila-34 | 5 | 1 |
| F.brevipila-35 | 5 | 3 |
| F.brevipila-38 | 14 | 5 |
| F.brevipila-42 | 3 | 1 |
| F.brevipila-43 | 12 | 5 |
| F.brevipila-44 | 5 | 3 |
| F.brevipila-48 | 13 | 7 |
| F.brevipila-49 | 6 | 3 |
| F.brevipila-50 | 14 | 5 |
| F.brevipila-51 | 4 | 1 |
| TOTAL | 258 |  |
